# Metronidazole or Cotrimoxazole Therapy Is Associated with a Decrease in Intestinal Bioavailability of Common Antiretroviral Drugs

**DOI:** 10.1371/journal.pone.0089943

**Published:** 2014-02-26

**Authors:** Flore Dossou-Yovo, Godefroy Mamadou, Imar Djibrine Soudy, Nicolas Limas-Nzouzi, Joe Miantezila, Jehan-François Desjeux, Bruno Eto

**Affiliations:** 1 Laboratoire de Biologie, CNAM, Paris, France; 2 University of Paris Diderot - Paris 7, Faculty of Medicine Xavier Bichat, Laboratory TBC TransCell-Lab, Paris, France; 3 Institut Universitaire des Sciences et Technique d’Abéché, N’Djamena, Tchad; 4 Académie Nationale de Médecine, Paris, France; Charité, Campus Benjamin Franklin, Germany

## Abstract

Metronidazole (MTZ) and Cotrimoxazole (CTX) are used in HIV/AIDS patients eligible for antiretroviral treatment. The objective of this animal study was to determine whether pre-treatment with antibiotics affects the intestinal bioavailability of Atazanavir (ATV) and Ritonavir (RTV). After oral administration of 1 mg MTZ and CTX for 7 days, the rat colonic mucosa were analyzed for mucus thickness or placed in Ussing chambers to measure ATV and RTV net transepithelial fluxes (J_net_). 1. In control rats, the mucus thickness was 43.3±7.6 µm and 40.7±6.9 µm, in proximal and distal colon, respectively. In proximal colon, the thickness was 57.2±8.8 and 58.2±6.9 µm after MTZ and CTX, respectively whereas in distal colon, the thickness was 121.1±38.4 and 170.5±35.0 µm (P<0.05) respectively. 2. Transepithelial conductance was reduced after MTZ or CTX in the proximal and distal colon. 3. In control, net ATV secretion was observed both in proximal (−0.36±0.02 µg.hr^−1^ cm^−2^) and distal colon (−0.30±0.08 µg.hr^−1^ cm^−2^). After MTZ and CTX, it was increased in the proximal colon by two 2 fold and 4 fold, respectively and in the distal colon by 3 fold and 5 fold, respectively. 4. In control, there was no net active RTV transport either in proximal (+0.01±0.01 µg.hr^−1^ cm^−2^) or distal colon (+0.04±0.01 µg.hr^−1^ cm^−2^). After MTZ and CTX, secretion was increased 5 fold and 10 fold, respectively, in the proximal colon and two fold and 5 fold, respectively in the distal colon (p<0.001). In conclusion, after MTZ and CTX therapy, the mucus layer was enlarged, passive permeability was decreased and ATV and RTV were actively secreted by the colonic epithelium suggesting that, in rat, the intestinal bioavailability of ATV and RTV is impaired after antibiotic therapy.

## Introduction

Atazanavir (ATV) is an azapeptide HIV-1 protease inhibitor (PI) and one of the antiretrovirals (ARV) used in second-line therapy in adults and adolescents with HIV infection according to the recommendations for a public heath approach of the World Health Organization (WHO)’s 2010 guidelines. The oral bioavailability of ATV is low. Clinically, it is generally co-administered with Ritonavir (RTV), which stimulates ATV oral bioavailability by inhibiting cytochrome P450 (CYP) 3A, and P-glycoprotein (Pgp) via the same metabolic pathway [1], [2].

Metronidazole (MTZ) and Cotrimoxazole (CTX) are two antibiotics commonly used in HIV/AIDS patients; in particular, in those with baseline CD4 counts close to that of patients eligible for antiretroviral treatment. CTX remains popular due to its low cost, effectiveness and familiarity among clinicians. It is the most common antibiotic prescribed to patients with urinary tract infections [3]. Other indications include treatment of infections caused by *Pneumocystis jiroveci* (*Pj*), *Toxoplasma gondii* (*Tg*), *Stenotrophomonas maltophilia* and community-associated methicillin-resistant *Staphylococcus aureus*. In addition, low-dose CTX is commonly used for prophylaxis against opportunistic Pj and Tg infections [4] among patients with depressed CD4 counts. A recent study showed that MTZ therapy is associated with an increase of luminal mucus thickness in rats [5] pointing to a potential deleterious effect on the drug’s intestinal bioavailability.

After oral administration, the intestinal bioavailability of solid forms depend on the aqueous solubility of the drug, the concentration of the dissolved drug and/or its its permeability through the mucosal intestinal membrane. The mucus gel layer that coats the luminal epithelial layer is an unstirred aqueous layer and one of the main limiting factors to intestinal permeability [6], [7], [8], [9]. This results in a decrease in drug concentration across the mucus thickness [10]. For example, a simultaneous decrease in luminal mucus and increase in intestinal permeability occurs during total parenteral nutrition [11] or following the administration of a mucolytic agent [7].

Thus, the aim of the study was to determine whether Metronidazole and Cotrimoxazole, that increase the mucus thickness, also alter the intestinal bioavailability of Atazanavir and Ritonavir, the most frequently prescribed antiretroviral drugs in first and second line therapy for Acquired Immune Deficiency Syndrome (AIDS). To do so, the 3 main parameters of in vitro intestinal epithelial bioavailability were measured, namely: (1) variation of the mucus thickness, (2) the passive transmucosal permeability through electrical conductance, and (3) active transport through the net transepithelial fluxes of Atazanavir and Ritonavir.

## Materials and Methods

### Chemicals

Atazanavir (Reyataz, Bristol-Myers Squibb Pharma) and Ritonavir (Norvir, Abbot Laboratories Limited), Trimethoprim-sulfamethoxazole or Cotrimoxazole (Bactrim, Roche, France) and Metronidazole (Flagyl, Sanofi-aventis, France) and Bumetanide (Sigma, St. Quentin-Fallavier, France) were commercially available. All other chemicals were analytic grade reagents.

### Antiretroviral Solution Preparation

Solutions were prepared according the method of Ribière [12]. The sample weight (equivalent to two or four tablets or capsules, accurately weighed) was placed directly into a graduated flask with a little Ringer’s solution, and magnetically stirred for 30 min. ATV was then diluted to the required volume with Ringer’s and RTV with methanol 5% - Ringer’s 95%. The solutions were centrifuged for 15 min. Fingerprints of each supernatant were performed with UV spectra for the control before storage at 18°C and use within one week.

### Animals

Mature male Sprague-Dawley rats, weighing 180–250 g, were obtained from Janvier SAS (Route des chênes, Le Genest-st-Isle, St Berthevin, France), housed in individual cages and fed with standard laboratory chow (UAR, Villemoisson s/Orge, France).

The study was conducted in accordance with the accepted principles outlined in the “Guide for the Care and Use of Laboratory Animals” prepared by the National Academy of Sciences and published by the National Institutes of Health, and all efforts were made to minimize animal suffering and the number of animals used. Ethics approval was obtained from Paris Diderot University - Paris 7.

The studies were conducted in fasted animals. Food was withdrawn 18 hours before the experiments, although they had access to drinking water. The animals were then killed by CO_2_ inhalation, and their colons were removed and rinsed free of intestinal content by flushing with ice-cold Ringer’s solution. The animals’ stomachs were empty. The issues were opened along the mesenteric border, and mounted as flat sheets between the two halves of acrylic Ussing chambers, as previously described. In this study, we selected the colon because CTX and MTZ are mainly used to target the colonic bacteria. In addition, it was reported that MTZ was found in human ascending colon after oral administration where it was partially metabolized [13]. The antibiotic treated animals received 1 mg CTX or 1 mg MTZ (per os) for 7 days prior to the experiment day.

### Thickness of the Mucus Layer

Colonic samples from the proximal and distal sections were used for these studies. The segments were immediately fixed in Carnoy’s solution for 24 h at 4°C. The specimens were then paraffin-embedded, and up to 50 cross sections of 5 µm thickness were cut per block and mounted on slides according to the classical histological technique [5]. ACombined Alcian blue (AB) and periodic acid Schiff reagent (PAS) protocole was used where the dyes are -specific for acidic and neutral mucins respectively. The principle consists demonstrating the presence of mucins and to clearly distinguish between acidic and neutral mucins. By first staining all acidic mucins with alcian blue, the acidic mucins, which are also PAS positive, will not react with the subsequent PAS reagent and only the neutral mucins are colored blue [14].

Paraffin was removed in xylene and sections were rehydrated for 3 min in 100%, 96% and 70% ethanol. Sections were stained with Alcian blue/periodic acid Schiff (AB/PAS) (1% AB 8GX in 3% acetic acid, pH 2.5) for 10 min. After washing in 3% acetic acid and deionized water, the sections were oxidized in 0.5% periodic acid for 5 min, and then washed with deionized water, dehydrated in 100% alcohol and mounted on slides with Eukitt (Kindler GmbH, Freiburg, Germany) to measure the adherent mucus layer thickness [15]. Slides were examined using a high-power oil immersion lens in a Leica microscope DFC 300FX. Images were taken with Leica FW 4000 software (Leica microsystemes SAS, Rueil-Malmaison, France). Mucus was the stained blue gel layer sandwiched between the epithelial area and the luminal contents.

### Transepithelial Electrical Conductance

The isotonic Ringer’s solution used throughout the experiments contained (in mM) 115 NaCl, 25 NaHCO_3_, 1.2 MgCl_2_, 1.2 CaCl_2_, 2.4 K_2_HPO_4_, and 0.4 KH_2_PO_4_. The pH was 7.40 at 37°C when bubbled with the 95% O_2_ - 5% CO_2_ mixture used to circulate the chamber fluid. Ringer’s solution was used in the two bathing reservoirs on each side of the colon defining the two compartments: mucosal (or luminal) and serosal (or blood side) compartments, separated by the colonic mucosa.

The spontaneous transmural electrical potential difference (PD) reflecting the asymmetry of electrical charges between the luminal and serosal colonic mucosa was measured via 3 M KCl solution in 4% (w/v) agar bridges. These bridges were placed on both sides of the tissue and connected to calomel half-cells, linked to a high-impedance voltmeter. PD was short-circuited and maintained at 0 mV throughout the experiment by a short-circuit current (*I*_sc_) via two stainless steel 316L working electrodes directly placed in each reservoir as described by Mathieu et al [16], in relation with an automatic voltage-clamp system (JFD-1V, Laboratoires TBC & Biomécatronics SAS, Ruitz, France).

Delivered *I*_sc_, corrected for fluid resistance, was recorded continuously on a computer with Biodaqsoft software (Laboratoires TBC & Biomécatronics SAS, Ruitz, France). The *I*_sc_ (in µA/cm^2^) represents the sum of the net ion fluxes transported across the epithelium in the absence of an electrochemical gradient (mainly Na^+^, Cl^−^, and HCO_3_^−^). The transepithelial electrical conductance (G_t_) was calculated according to Ohm’s law. G -reverse of resistance a permeability parameter was expressed in mS/cm^2^.

### Atazanavir and Ritonavir Transepithelial Fluxes

At the steady state of electrical parameters, tissues were paired according to their conductance value (±20%). After *I_sc_* stabilised, Ringer’s solution containing Atazanavir (ATV, 1 mg) and Ritonavir (RTV, 1 mg) were then introduced at the same time into the serosal or the mucosal compartment of paired tissues. A 1-ml sample from the opposite compartment was withdrawn at 0, 60 and 120 min and replaced by 1 ml of Ringer’s solution at 37°C. The ATV and RTV samples were then diluted with Ringer’s solution to 3 ml for UV measurements at 237 nm and 240 nm, respectively, as described by Ribiere et al [12]. The effects of pre-treatment with MTZ or CTX on unidirectional mucosal-to serosal and serosal-to-mucosal ATV and RTV fluxes were determined during the steady state of transport (60–120 min). The net ATV and RTV fluxes were the differences between the opposite unidirectional fluxes obtained on paired tissues. Positive values indicate absorption from mucosa (luminal side) to serosa (blood side) across the epithelium while negative values indicate secretion.

The relationship between transepithelial electrical conductance (Gt) and mucus thickness (MT) was analyzed using the “generate and stimulate curves” function of the Graphpad Prism 5 program (San Diego, CA, USA).

### Statistics

The data are expressed as a mean ± standard error (SE), *n* = number of tissues of at least 3 rats. The statistical analyses were obtained by two way analysis of variance (ANOVA), using as source of variation treatment (MTZ, CTX) and tissue (distal and proximal colon) followed by the Bonferroni post-tests (Graphpad software for Windows version 5. Graphpad, San Diego, CA, USA). P<0.05 was considered as significant.

## Results

### Mucus Thickness (MT)

**Figure 1:**
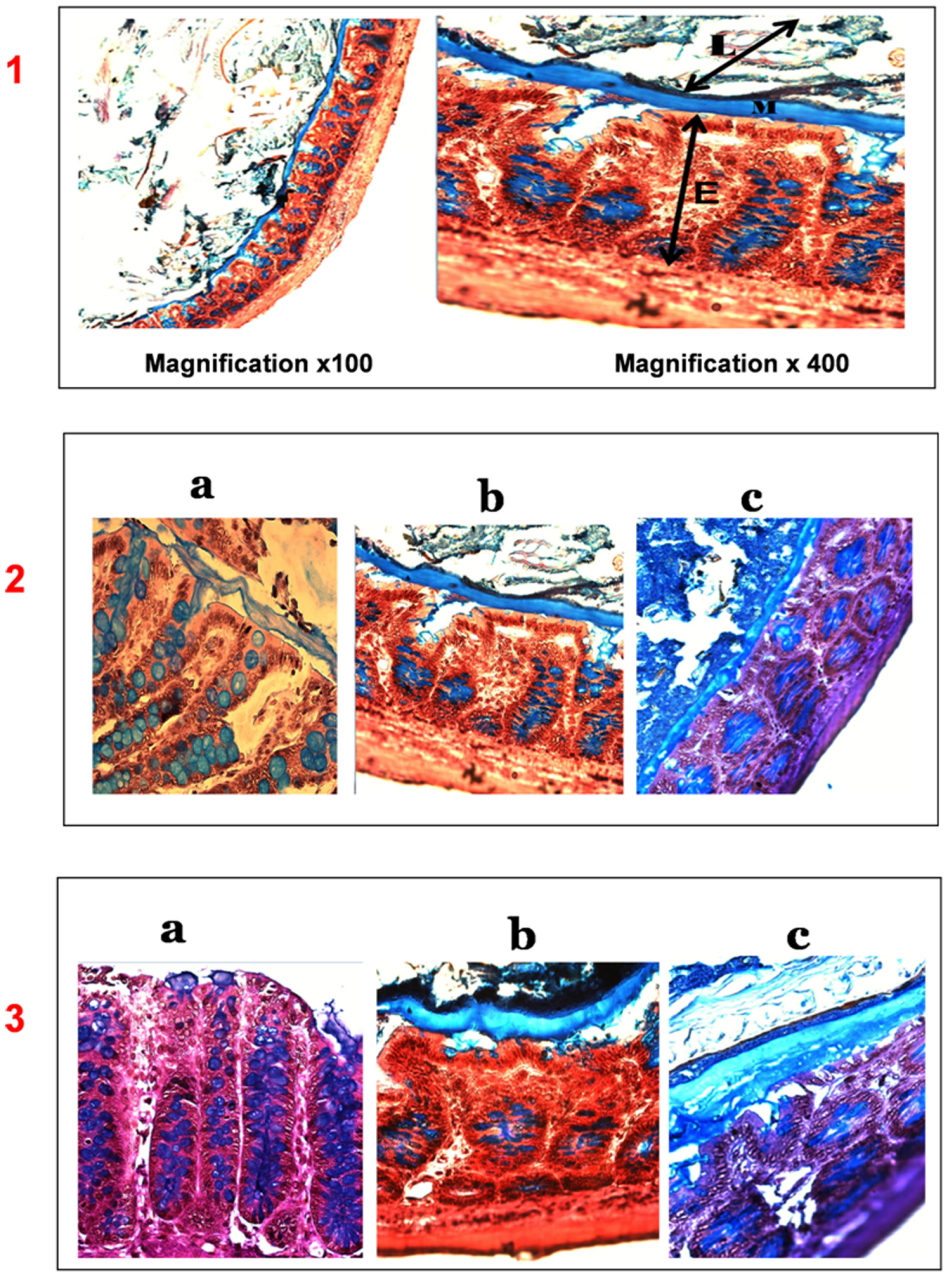
Thickness of the total mucus layer in colonic mucosa in proximal 2) and distal segments 3) of control a), Metronidazole b) and Cotrimoxazole-treated rats c), AB/PAS staining. 1) Magnification x 100 and x 400. The total mucus thickness (**M**) was measured as a continuous layer between the luminal surface (**L**) and epithelium (**E**). Increased thickness of the mucus layer was observed in MTZ and CTX treated rats (**b**, **c**). Goblet cells are filled with mucus (blue arrows) either in the control or the MTZ and CTX treated group. Occasionally, a separation could be observed between the mucus layer and the epithelial surface, and may be due to the partial mucus shrinkage during the histological procedure [37].

**Figure 2:**
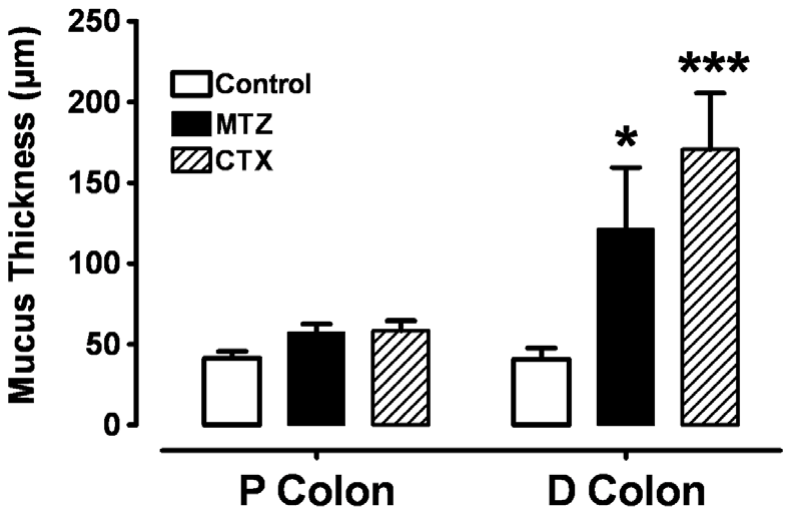
Mucus thickness of colonic mucus (mean ± SE) after 1 week pre-treatment with 1 mg of Cotrimoxazole (CTX) or 1 mg of Metronidazole (MTZ), compared to control in proximal and distal colon. *P<0.05 and ***P<0.001, n = 24 tissues from 7 rats.

Pre-treatment of animals with antibiotics resulted in an increase in intestinal thickness of mucus in proximal and distal colon (fig. 1 and 2). In the proximal colon, after one week of MTZ or CTX treatment the thickness of mucus was 57.2±8.8 µm and 58.2±6.9 µm, respectively, vs. 43.3±7.6 µm in controls, but the increase was not statistically significant. In the distal colon, it was 121.1±38.4 µm and 170.5±35.0 µm respectively, vs. 40.6±6.9 µm in controls.

### Transepithelial Electrical Conductance

**Figure 3:**
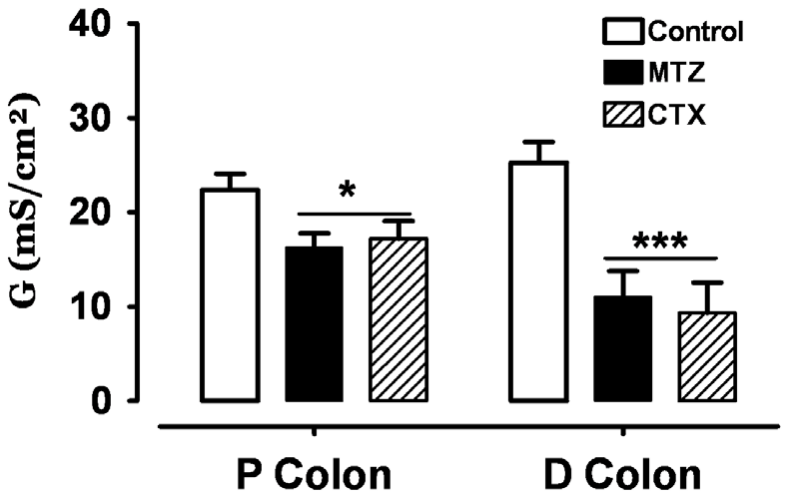
Transepithelial electrical conductance (G_t_) (mean ± SE) after 1 week of pre-treatment with 1 mg of Cotrimoxazole (CTX) or 1 mg of Metronidazole (MTZ) compared to controls, in proximal (A) and distal (B) colon. *p<0.05, ***P<0.001, n = 24 tissues from 7 rats.

Transepithelial conductance (G_t_) was decreased after one week of treatment with MTZ or CTX in the proximal and distal colon. In controls, the conductance (G_t_) was 22.4±1.7 in proximal colon and 25.3±3.7 mS/cm^2^ in distal colon (fig. 3); whereas one week after MTZ or CTX treatment the conductance was more reduced in the distal colon than in proximal colon. In the proximal colon it was 16.3±1.7 mS/cm^2^ with MTZ and 17.2±1.8 mS/cm^2^ with CTX. In the distal colon, it was 11.1±3.8 with MTZ and 9.3±3.3 mS/cm^2^ with CTX.

### Relationship between Transepithelial Electrical Conductance (Gt) and Mucus Thickness (MT)

**Figure 4:**
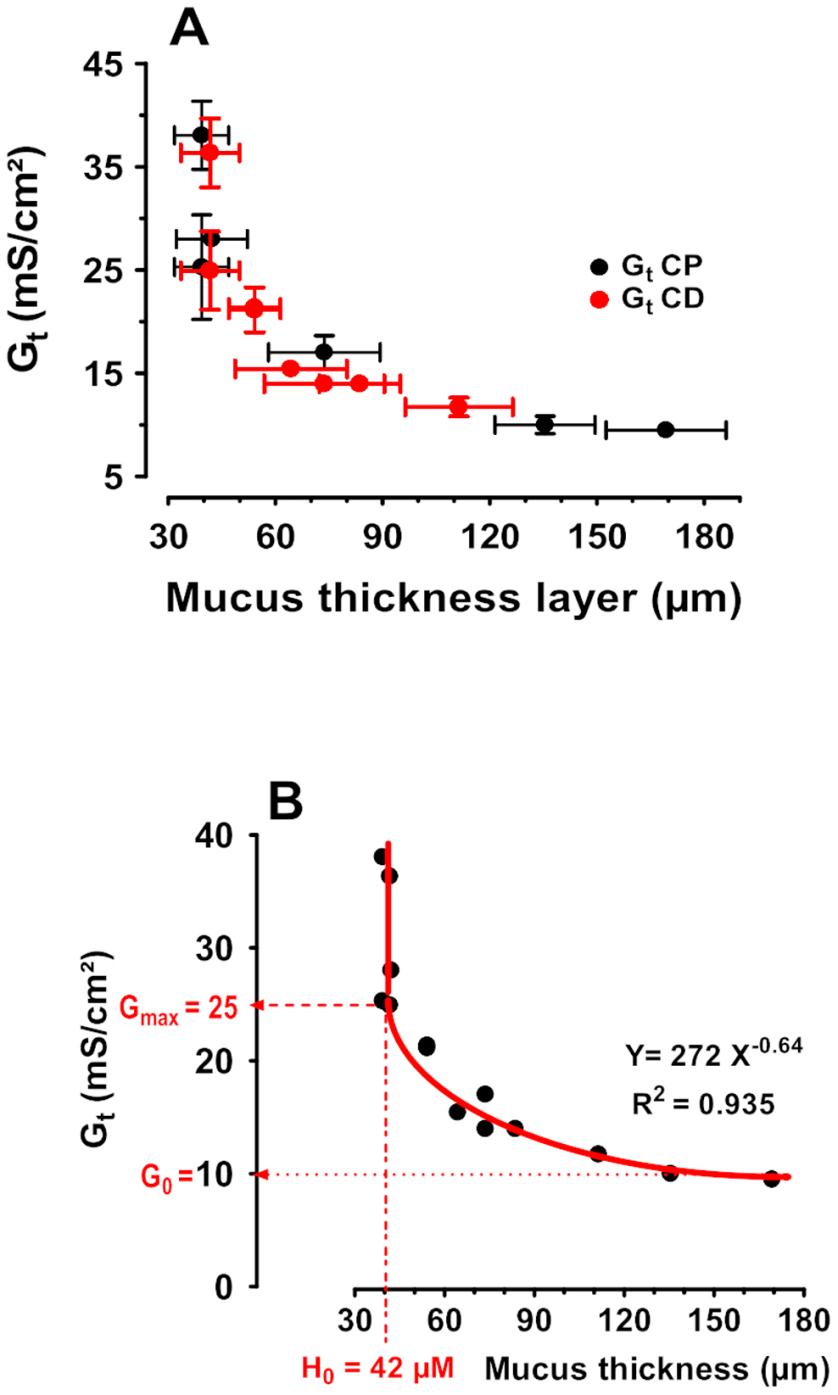
Relationship between Mucus thickness (in µm) and Transepithelial electrical Conductance G_t_ (in mS/cm^2^). The data were obtained from the means of at least n = 13 tissues from 7 rats. Closed black dots indicate proximal colon and closed red dots distal colon (A). The relationship between Gt and mucus thickness (ML) were analyzed by nonlinear regression (curve fit) using the power series equation Y = AX^b^ (B). r^2^ was 0.94. With 95% confidence limit if of A of 168.6 to 374.6 and B of −0.73 to −0.55, with covariance Matrix A and B = −0.99. Below the threshold of 42 µm the conductance (an index of ionic permeability) remained constant 25 mS/cm^2^. In contrast, the conductance decreased with mucus thickness down to a limit value of 10 mS/cm^2^.

The relationship was highly significant (r^2^ = 0.94). Interestingly, the data obtained on proximal and distal colon did not depart from each other (fig. 4A). Thus, the analysis was performed with the combined data (fig. 4B). The best fit is a power series relationship Y = AX^B^, (G_t = _272 MT^−0.64^).

### ATV and RTV Net Transepithelial Fluxes

**Figure 5:**
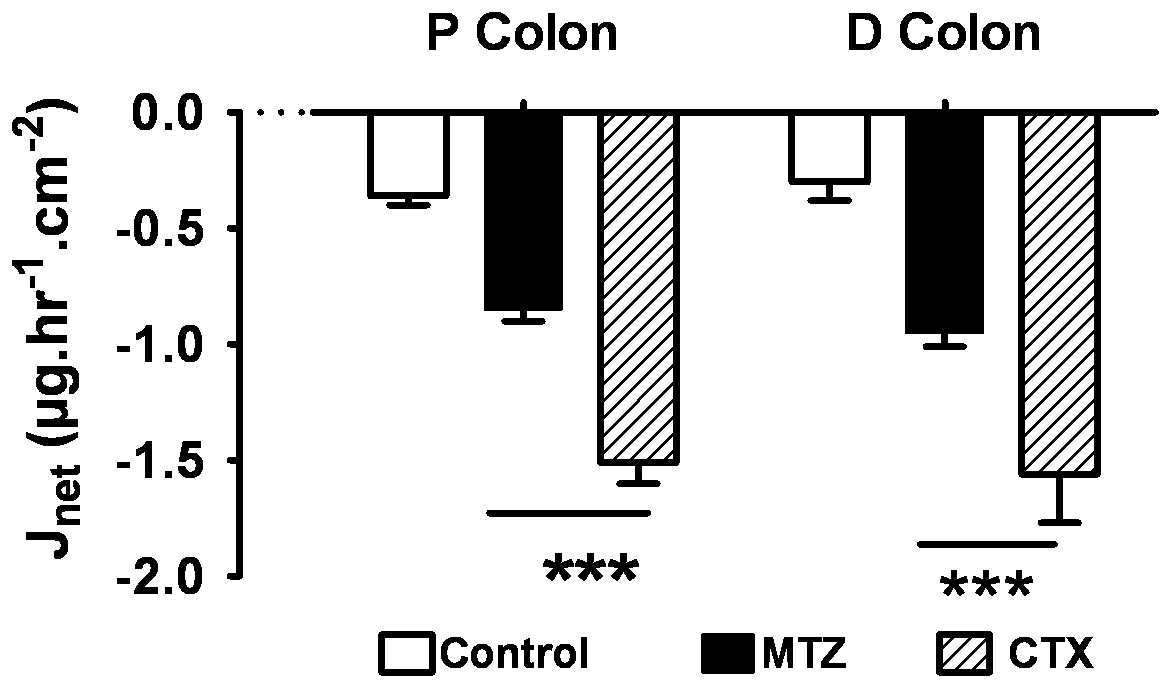
ATV transepithelial net fluxes (J_net_) (mean ± SE) after 1 week of pre-treatment with 1 mg of Cotrimoxazole (CTX), or 1 mg of Metronidazole (MTZ) compared to controls, in proximal and distal colon. ***p<0.001, n = 12 tissues from 4 rats. Negative J_net_ indicates secretion from serosal (blood) to mucosal (luminal) side.

In proximal and distal colon, pre-treatment of rats with both MTZ and CTX reduced the transepithelial intestinal bioavailability of ATV. In proximal colon, there was a net secretion (−0.36±0.02 µg.hr^−1^⋅cm^−2^) in controls. Secretion was significantly increased after MTZ or CTX: −0.85±0.05 µg.hr^−1^⋅cm^−2^ and −1.50±0.09 µg.hr^−1^⋅cm^−2^, respectively (P<0.001) (fig. 5). Similarly, in the distal colon, there was a net secretion in controls (−0.30±0.08 µg.hr^−1^⋅cm^−2^). This was significantly increased after MTZ or CTX: −0.95±0.06 µg.hr^−1^⋅cm^−2^ (p<0.05) and −1.56±0.31 µg.hr^−1^⋅cm^−2^ (P<0.001), respectively (fig. 5).

**Figure 6:**
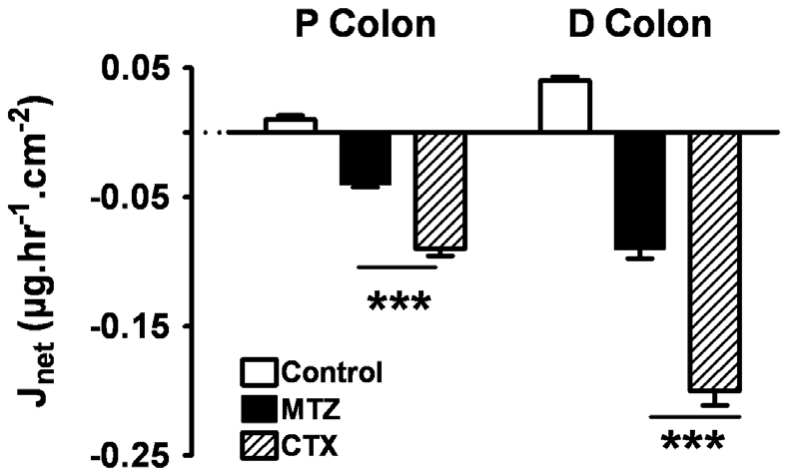
RTV transepithelial net fluxes (J_net_) (mean ± SE) after 1 week of pre-treatment with 1 mg of Cotrimoxazole (CTX) or 1 mg of Metronidazole (MTZ) compared to controls, in proximal and distal tissues. ***p<0.001, n = 12 tissues of 4 rats.

RTV secretion was also stimulated although to a lesser extent compared to ATV. In the proximal colon, in controls, there was no net transport (+0.01±0.01 µg.hr^−1^⋅cm^−2^). A significant secretion was observed after MTZ or CTX: −0.04±0.01 µg.hr^−1^⋅cm^−2^ (P<0.001) and −0.09±0.02 µg.hr^−1^⋅cm^−2^ (P<0.001), respectively (fig. 6).

In the distal colon, in controls, there was almost no net transport (+0.04±0.01 µg.hr^−1^⋅cm^−2^). A significant secretion was observed after MTZ or CTX: −0.09±0.03 µg.hr^−1^⋅cm^−2^ (P<0.001) and −0.20±0.04 µg.hr^−1^⋅cm^−2^, (P<0.001)^,^ respectively (fig. 6).

## Discussion

The present study confirms that 7 days of 1 mg Cotrimoxazole (CTX) or metronidazole (MTZ) therapy induced an increase in the thickness of the mucus gel layer that coats the luminal surface of rat colonic epithelium [5]. In addition, it indicates that the increased thickness of the mucus layer was associated with a decrease in transepithelial conductance, a non-specific marker of small water-soluble molecule permeability. Antibiotic therapy was also associated with an increase in active secretion of both antiretroviral drugs to the luminal side, although it was more pronounced with ATV than with RTV. Taken together, the present results are consistent with a decrease in intestinal colonic mucosal bioavailability of two major anti-retroviral drugs after 7 days of therapy with two frequently used antibiotics CTX and MTZ. This could be explained, at least in part, by an increase in thickness of the mucus covering the epithelial layer contributing to a decrease in passive diffusion and by the stimulation of an active drug secretion by the epithelium.

The colonic epithelium is covered with mucus, a gel-like fluid containing mainly water (90–98%) and mucin (2–5%). This aqueous gel-like matrix forms an unstirred water layer (UWL) creating an aqueous diffusion barrier that hampers drug permeation [17]. For many solutes, crossing the mucus layer is associated with a reduction in effective diffusion coefficients, and with retardation of passive diffusion solute fluxes [18], [19]. Variability in small solute passive diffusion is frequently explored by measurements of an alteration of transepithelial electrical conductance. This method is based on the observation of a linear relationship between conductance and transepithelial flux of small water-soluble molecules, including sodium chloride (MW 58), xylose (MW 150), mannitol (MW 182), or polyethylene glycol (MW 2000) [20], [21]. Thus, the present finding of a significant reduction in conductance after 7 days of antibiotic exposure is consistent with a reduction in active ATV and RTV permeability.

In addition, from the relationship between mucus thickness and conductance it can be postulated that the passive permeability of many drugs of small molecular weight may be altered by agents that modulate mucus thickness. Below approx. 40 µm, the conductance remains constant, suggesting that the remaining mucus is part of the epithelial functional entity. In addition, when the mucus layer is thicker than 40 µm the conductance is reduced following the equation G_t = _272 MT^−0.64^. In the present study, increasing this part of the mucus layer by threefold, from 40 to 160 µm, was associated with a conductance decrease of more than half. It remains to be seen if such a relationship is also present with other small-molecular-weight water-soluble drugs.

The observed increased active secretion of ATV and RTV after CTX and MTZ further suggests that the antibiotics may not only decrease passive diffusion but also stimulate active transport system(s), both contributing to decreasing the bioavailability of the retroviral drugs. The Ussing chamber apparatus was essentially designed to study active transport across an epithelial layer [22], including the colonic epithelial layer [23]. In controls, ATV was actively secreted into the luminal compartment and no active transport of RTV was observed. In contrast active secretion was greatly increased after antibiotic treatment, although less pronounced with RTV than with ATV. This finding suggests that the cellular epithelial transporters that secrete xenobiotics are stimulated by antibiotics. Part of the observed secretion may be related to a decrease in ARV drug concentration at the surface of the epithelial cells due to the increased UWL, depending on the Km of the specific transporter(s). It also points to the potential stimulation of the ATP-binding cassette transporters that are known to secrete antiretroviral drugs [24], [25]. The in vivo oral bioavailability of ATV is low. In patients, it is generally co-administered with RTV because RTV stimulates oral ATV bioavailability by inhibiting cytochrome P450 (CYP) 3A, and P-glycoprotein (Pgp) via the same metabolic pathway [1], [2]. In addition, it has also been reported that chronically used RTV is not only a powerful protease inhibitor, but an efficient inducer of CYP3A and Pgp [26], [27]. The opposing actions of RTV and ATV on bioavailability modulators may be further altered by the effects of commonly used of antibiotics. In this animal study ATV was used concomitantly with RTV in order to mimic clinical usage.

We did not study other mechanisms by which antibiotics could alter the intestinal bioavailability of anti-retroviral drugs. However, several additional mechanisms may be involved: For example, the bacteria that are retained in the mucus may also directly influence epithelial properties. In the colon, the mucus layer provides a hospitable environment for the microbiota, where the host and bacteria benefit from a symbiotic relationship [28], [29], [30]. Recently, an increase in bifidobacteria and enterobacteria was found in metronidazole-treated rats compared with control rats [5]. This was associated with a decrease in oxidative stress, a condition that is associated with alterations in intestinal epithelial transport properties [31]. In rat, gut microbiota and dietary composition have been shown to affect the secretory pattern of intestinal mucins. In the small intestine, the effect of diet was more pronounced, whereas in the large intestine the microbiota was the main modulator [5], [28], [32], [33]. Therefore, variations in terms of mucus thickness may not only be linked to internal physiological factors but also to external factors. In this study, the externally acting factors were the antibiotics (CTX and MTZ).

In addition, impaired or aberrant mucus production is often associated with an inflammatory reaction, including in inflammatory bowel diseases [34], [35]. In turn, mucosal inflammation is associated with altered intestinal epithelial permeability properties [34], [36]. In the present study, we did not specifically explore the relative roles of oxidative stress and immune response in the observed decreased passive diffusion and increased active transport.

In conclusion, this study clearly shows that CTX and MTZ, often prescribed to HIV patients in Sub-Saharan Africa, as a prophylaxis of opportunistic infections, induced a reduction the *in vitro* bioavailability of ATV and RTV in rat colon. Complementary studies in humans are necessary to confirm this observation. This study suggests that the reduction of the oral bioavailability of ARVs must be taken in account after CTX and MTZ. The reduction of systemic drug concentrations, particularly proteases inhibitors, during treatment can be one of the factors favouring HIV drug resistance, particularly in developing countries. If true, substitution of CTX or MTZ by other molecules, including immuno-modulating medicines may be an alternative in the prophylaxis of opportunistic infections.
